# Optimization of Ultrasonic-Assisted Extraction of Flavonoid Compounds and Antioxidants from Alfalfa Using Response Surface Method

**DOI:** 10.3390/molecules200915550

**Published:** 2015-08-26

**Authors:** Chang-Liang Jing, Xiao-Fang Dong, Jian-Ming Tong

**Affiliations:** Institute of Animal Sciences, Chinese Academy of Agricultural Sciences, Beijing 100193, China; E-Mails: jingchangliang2008@163.com (C.-L.J.); tjm606@iascaas.net.cn (J.-M.T.)

**Keywords:** alfalfa, flavonoid compounds, ultrasonic-assisted extraction, response surface methodology, antioxidant capacity

## Abstract

Ultrasonic-assisted extraction (UAE) was used to extract flavonoid-enriched antioxidants from alfalfa aerial part. Response surface methodology (RSM), based on a four-factor, five-level central composite design (CCD), was employed to obtain the optimal extraction parameters, in which the flavonoid content was maximum and the antioxidant activity of the extracts was strongest. Radical scavenging capacity of the extracts, which represents the amounts of antioxidants in alfalfa, was determined by using 2,2′-azino-bis (3-ethylbenzothiazoline-6-sulphonicacid) (ABTS) and 2,2′-diphenyl-1-picrylhydrazyl (DPPH) methods. The results showed good fit with the proposed models for the total flavonoid extraction (R^2^ = 0.9849), for the antioxidant extraction assayed by ABTS method (R^2^ = 0.9764), and by DPPH method (R^2^ = 0.9806). Optimized extraction conditions for total flavonoids was a ratio of liquid to solid of 57.16 mL/g, 62.33 °C, 57.08 min, and 52.14% ethanol. The optimal extraction parameters of extracts for the highest antioxidant activity by DPPH method was a ratio of liquid to solid 60.3 mL/g, 54.56 °C, 45.59 min, and 46.67% ethanol, and by ABTS assay was a ratio of liquid to solid 47.29 mL/g, 63.73 °C, 51.62 min, and 60% ethanol concentration. Our work offers optimal extraction conditions for total flavonoids and antioxidants from alfalfa.

## 1. Introduction

Alfalfa (*Medicago sativa* Linn) is a well-known Chinese medicine herb that is distributed widely in China. It has been used historically as a medicine to treat coughs and digestive problems and is used as a dietary supplement in different forms (tablets, powders, and tea) to benefit both humans and animals [1]. Numerous reports have proved that alfalfa has immunostimulatory, antioxidant, pharmacological, and antidiabetic activity [2,3], which are mainly attributed to the phytochemicals derived from the plant such as flavonoids [4], polysaccharides [5], and saponins [6].

Flavonoids, as phenolic compounds, have shown significant antioxidant activity both *in vitro* and *in vivo* [7,8]. In several studies, different amounts of flavonoids have been found in alfalfa tissues, which indicated that it may be utilized as a potential natural antioxidant compound [9,10,11,12]. Alfalfa may be a promising resource for flavonoids in the future. In order to explore the efficient utilization of flavonoids from alfalfa, more bioactivities should be studied and the extraction technology is deserved to develop and optimize.

Up to now, several conventional extraction techniques have been used to extract flavonoids like hot water extraction [9], alkaline extraction [13], microwave-assisted extraction [14], supercritical fluid extraction accelerated solvent extraction [15], and ultrasonic-assisted extraction [16,17]. Among these, ultrasonic-assisted extraction has been successful and widely used owing to its high efficiency, time-saving, simplified manipulation, and is environmental-friendly [18,19].

Response surface methodology is a collection of statistical and mathematical techniques for optimization study [20] and is widely used to optimize conditions for extracting active compounds from herbs [21,22]. The main advantage of RSM is to reduce the number of experiments necessary to conduct the research. Thus, it leads to time-saving, as well as a decrease in the consumption of reagents and materials [23].

To date, there is little information about optimizing the ultrasonic-assisted extraction of total flavonoids from alfalfa using central composite rotatable response surface design (CCRD). The objective of this study is to establish the optimized parameters of ultrasound-assisted extraction for the flavonoids from alfalfa. At the same time, the particular goal is to retain optimal 2,2′-diphenyl-1-picrylhydrazyl (DPPH) and 2,2′-azinbis (3-ethylbenzothiazoline-6-sulphonic acid) (ABTS) radical-scavenging capacities of the extracts for development and application of the source as a novel potential antioxidant.

## 2. Results and Discussion

### 2.1. Single-Factor Experiments

Single-factor experiments were used to determine whether the ratio of liquid to solid, extraction temperature, extraction time, and ethanol concentration could be optimized for total flavonoid content (TFC) and antioxidant extraction. Then, the appropriate ranges of extraction variables were determined.

#### 2.1.1. Effects of Different Ratio of Liquid to Solid

**Figure 1:**
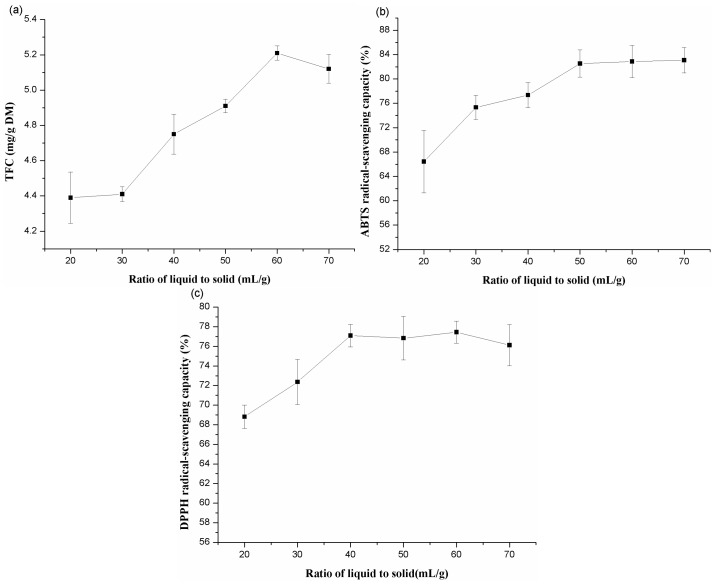
Effects of liquid to solid ratio on the extraction yield of total flavonoids (**a**); ABTS (**b**) and DPPH (**c**) radical-scavenging capacities of extracts from alfalfa.

The ratio of liquid to solid is an important parameter that can influence the extraction efficiency [24,25]. Numerous studies have demonstrated that a suitable ratio of liquid to solid could contribute to the extraction of flavonoids [26,27]. Total flavonoids in a material could be completely extracted when the ratio of solvent to material is at an appropriate extent. In the present study, the ratio of liquid to solid was performed at 20, 30, 40, 50, 60, and 70 mL/g when the other reaction conditions were as follows: extraction time 60 min, extraction temperature 60 °C, and ethanol concentration 40%, respectively. As shown in Figure 1, the yield of total flavonoids increased greatly when the ratio increased from 20:1 to 60:1 (mL/g), and then showed a decrease when the ratio of liquid to solid increased beyond 60:1 (mL/g). ABTS radical-scavenging capacities had obviously increased with the increase of the ratio from 20:1 (mL/g) to 50:1 (mL/g) and then it increased slightly. DPPH radical-scavenging capacity was parabolic with a maximum value when the ratio of solvent to material reached 60:1 (mL/g).

The result shows that a suitable volume of solvent contributed to the yield of flavonoids and antioxidant compounds. We found that the lower the ratio of liquid to solid resulted in lower yield of TFC until it reached 60:1 (mL/g). This may be attributable to the suitable volume of solvent which could fully dissolve out the flavonoids from the material [28]. When the ratio exceeded 60:1 (mL/g), more impurities were dissolved out which hindered the dissolution of flavonoids. Antioxidant capacities may be due to both scavenging of ABTS radicals and DPPH radicals. The optimum ratio of liquid to solid for antioxidant capacity, measured with ABTS and DPPH, peaked at around 50:1 and 60:1 (mL/g), respectively. Results over the above, the ratio of liquid to solid of 30:1~70:1 (mL/g) was favorable in the present work.

#### 2.1.2. Effects of Different Extraction Temperature

**Figure 2:**
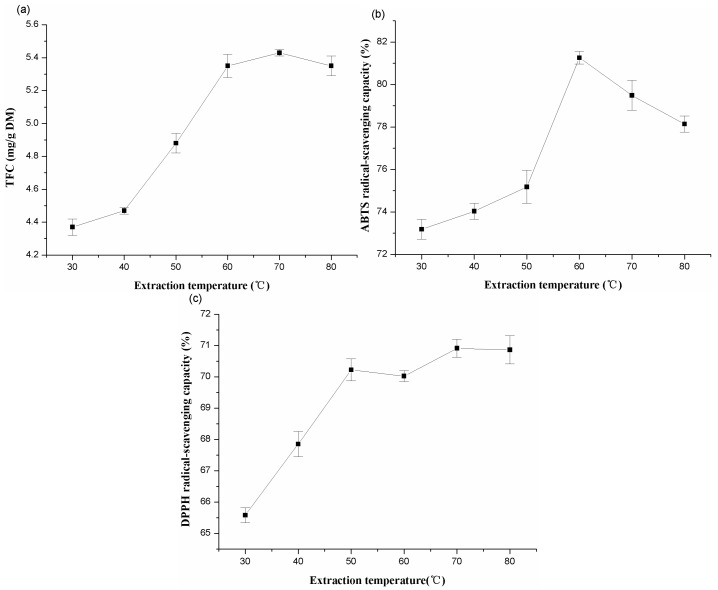
Effects of extraction temperature on the extraction yield of total flavonoids (**a**); ABTS (**b**) and DPPH (**c**) radical-scavenging capacities of extracts from alfalfa.

Extraction temperature would influence the molecular movement. Heat could accelerate large amounts of compound dissolution [29]. In this experiment, different temperatures (30, 40, 50, 60, 70, and 80 °C) were chosen to study the effects on TFC, ABTS, and DPPH radical-scavenging capacities of the extracts from alfalfa, while other extraction conditions were the ratio of liquid to solid (50:1, mL/g), extraction time of 60 min, and ethanol concentration at 40%. As shown in Figure 2, when the temperature varied from 30 to 80 °C, the optimal yield, in regards of TFC, ABTS, and DPPH radical-scavenging capacity were at 70, 60, and 70 °C, respectively.

Increased temperature led to an increase of molecular movement, which accelerated the dissolution of flavonoids from plant cells [25]. In this study, as Figure 2 depicted, the reason may be due to the suitable temperature making the increase of solubility and decomposition of plant cells, thus, favored the release of flavonoids from the material. Similar results have also been reported for the extraction of anthraquinones from roots of *Morinda citrifolia* [30]. However, elevated temperature would lead to the denaturation of the thermo-sensitive antioxidants, which might be more appropriate to mobilize at lower temperature [31]. Therefore, the extraction temperature of 60 °C was adopted for the subsequent RSM.

#### 2.1.3. Effects of Different Extraction Time

**Figure 3:**
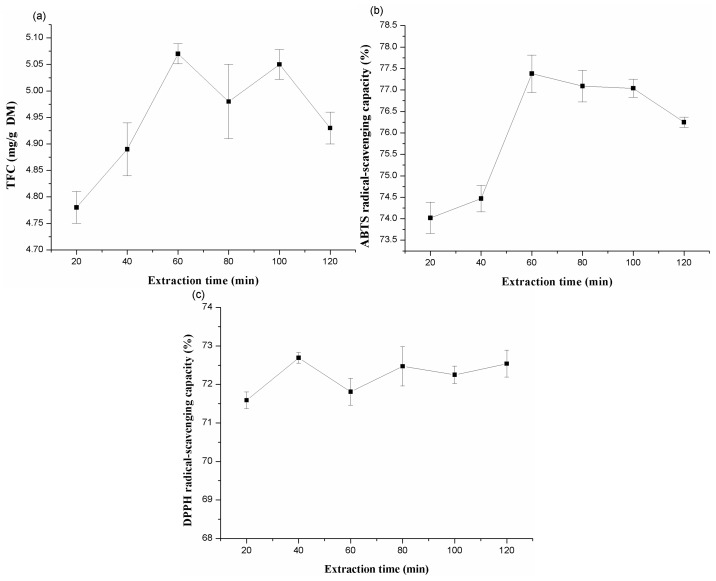
Effects of extraction time on the extraction yield of total flavonoids (**a**); ABTS (**b**) and DPPH (**c**) radical-scavenging capacities of extracts from alfalfa.

The extraction yield of flavonoids and the scavenging capacity of ABTS and DPPH radicals by different extraction time are shown in Figure 3, when the other three factors (liquid to solid ratio, extraction temperature, and ethanol concentration) were fixed at 50:1 (mL/g), 60 °C, and 40%, respectively. As shown in Figure 3, the extraction time affected the yield of total flavonoids significantly, and declined with the further increase in the extraction time when it reached the maximum values, 60 min and 100 min, respectively. However, there were little differences observed in yield of total flavonoids over 60 min and 100 min. This phenomenon may be due to different degrees of phenolic polymerization and the interaction of constituents in the extract, which leads to equilibrium being reached between the bulk solution and the solution in the material reached at different times [32]. Thus, an extraction time of 60 min was fixed for the next step, since longer times account for more energy and may decompose recovered compounds [33].

The optimum extraction time to get the highest scavenging capacity of ABTS radicals was at 60 min, and decreased thereafter. Extracts obtained at 40 min had a greater scavenging capacity for DPPH radicals. This may be due to the antioxidant compounds in the extracts obtained at different times showed differences by employing DPPH and ABTS scavenging assays, which was similar with previous reports [34,35,36].

#### 2.1.4. Effects of Different Ethanol Concentration

An elementary aspect in the choice of solvent is “like dissolves like”, which indicated the analyte of interest should have high solubility in the selected solvents [37]. Using a mixture of ethanol and water on the recoveries of phenols and flavonoids from different herbs has been discussed in some studies [38,39]. This is due to the wide range of phenol compounds that can dissolve in the aqueous ethanol mixture [40]. Furthermore, economy, safety, and sustainability are also important aspects in the choice of solvent. In regard to the solvent selected in the extraction, ethanol is a more suitable polar modifier due to its volatility. Moreover, ethanol has been studied as one of the most environmentally-friendly solvents and it is recognized as safe according to the European Food Safety Authority (EFSA) and FAO/WHO Expert Committee on Food Additives [41,42,43].

**Figure 4:**
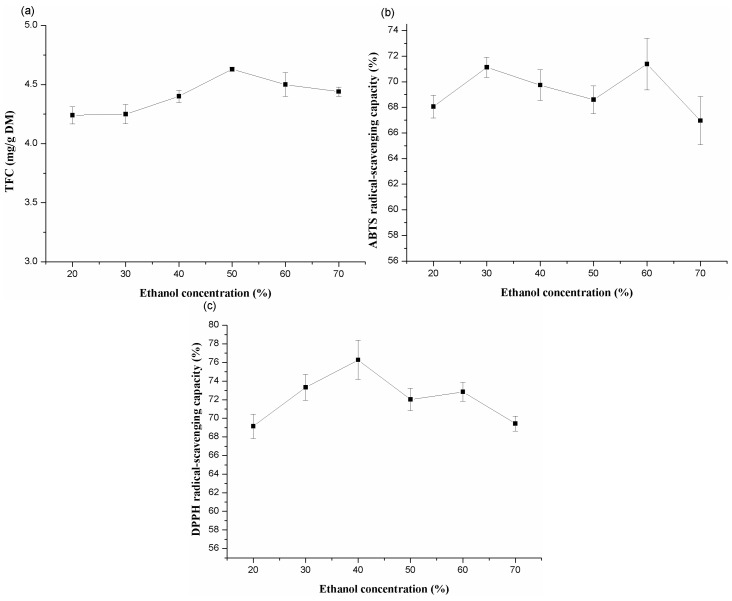
Effects of ethanol concentration on the extraction yield of total flavonoids (**a**); ABTS (**b**) and DPPH (**c**) radical-scavenging capacities of extracts from alfalfa.

To investigate the effect of ethanol concentration on the yield of TFC, ethanol concentrations of 20%, 30%, 40%, 50%, 60%, and 70% have been used. Figure 4 depicted of yield of flavonoids and the antioxidant capacities against ABTS and DPPH radicals. It showed that the yield of flavonoids increased as the ethanol concentration increased from 20% to 50%; after this point, it decreased considerably. ABTS radical-scavenging capacities initially increased and peak at 30% ethanol, then the other peak was reached at 60% ethanol. DPPH radical-scavenging capacity reached the peak around 40% ethanol. Therefore, 20%–60% ethanol concentrations were adopted for the next extraction.

Extracts may be present in different products under different extraction conditions [44]. Solvents exhibit different extraction capabilities due to the different polarity and chemical structure of the extracts. Previous studies demonstrated that a binary solvent system was superior to a mono-solvent system with respect to extracting phenolic compounds [45,46]. In this study, the maximum yield of flavonoids was observed at 60% ethanol, which suggests that the forms of flavonoids in alfalfa are highly soluble in this concentration and the differences in the yields from alfalfa may be assigned to the different polarity and chemical composition of flavonoids.

Antioxidant capacity of the extracts is largely dependent on the solvent polarity due to the different potential of compound polarity [9]. In this study, extracts from lower ethanol concentrations appeared higher DPPH (40%, *v*/*v*) and ABTS (30%, *v*/*v*) radical-scavenging capacities. However, the maximum yield of flavonoids obtained at ethanol concentration of 50%, which indicated that the flavonoid does not incorporate all the antioxidant compounds that are present in the extracts.

### 2.2. Optimization by RSM

#### 2.2.1. Fitting the Model

In the present study, central composite rotatable design (CCRD) was used to optimize the extraction parameters with response surface methodology. All the response values obtained from the 30-run-experiment and predicted data were shown in Table 1. The similarity between the experimental and predicted values shows the accuracy of the model. Maximum yield of TFC was recorded during Run No. 26 at 6.28 mg RE/g, and maximum radical-scavenging capacity of ABTS and DPPH was recorded at 87.04% and 81.09% during Run No. 24 and Run No. 10, respectively.

**Table 1:** Coded and real levels of the operational parameters and experimental and predicted values for different levels of experiment design.

Run	X_1_(Rls) ^a^	X_2_(Et) ^b^	X_3_(T) ^c^	X_4_(Ec) ^d^	TFC (mg RE/g)		ABTS (%)		DPPH (%)	
					exp	pred	exp	pred	exp	pred
1	40(−1)	50(−1)	40(−1)	30(−1)	4.76	4.81	76.30	77.60	68.98	69.00
2	60(1)	50(−1)	40(−1)	30(−1)	5.40	5.46	78.85	79.01	75.03	74.96
3	40(−1)	70(1)	40(−1)	30(−1)	4.98	5.01	78.96	79.08	75.15	74.57
4	60(1)	70(1)	40(−1)	30(−1)	5.39	5.45	79.57	76.69	74.07	73.93
5	40(−1)	50(−1)	80(1)	30(−1)	4.98	4.98	80.04	80.12	70.38	70.79
6	60(1)	50(−1)	80(1)	30(−1)	5.25	5.28	84.38	84.51	73.57	73.05
7	40(−1)	70(1)	80(1)	30(−1)	4.94	4.92	80.76	80.98	76.15	76.85
8	60(1)	70(1)	80(1)	30(−1)	5.00	5.01	83.99	84.57	72.94	72.49
9	40(−1)	50(−1)	40(−1)	50(1)	5.35	5.41	83.10	82.85	71.86	72.24
10	60(1)	50(−1)	40(−1)	50(1)	6.07	6.06	82.02	82.06	81.09	80.04
11	40(−1)	70(1)	40(−1)	50(1)	5.89	5.82	84.36	84.50	75.70	75.87
12	60(1)	70(1)	40(−1)	50(1)	6.19	6.26	82.68	82.92	77.55	77.06
13	40(−1)	50(−1)	80(1)	50(1)	5.84	5.75	81.13	81.28	72.46	72.25
14	60(1)	50(−1)	80(1)	50(1)	6.00	6.04	83.27	83.47	75.83	76.33
15	40(−1)	70(1)	80(1)	50(1)	5.88	5.89	82.15	82.31	76.36	76.35
16	60(1)	70(1)	80(1)	50(1)	6.05	5.97	84.41	83.70	74.20	73.82
17	30(−2)	60(0)	60(0)	40(0)	5.24	5.27	79.40	78.89	73.36	72.71
18	70(2)	60(0)	60(0)	40(0)	6.08	6.01	81.77	81.69	75.06	76.14
19	50(0)	40(−2)	60(0)	40(0)	5.53	5.48	82.29	81.85	73.20	73.25
20	50(0)	80(2)	60(0)	40(0)	5.61	5.62	83.71	83.56	75.94	76.32
21	50(0)	60(0)	20(−2)	40(0)	5.51	5.41	77.83	77.35	75.13	75.79
22	50(0)	60(0)	100(2)	40(0)	5.23	5.30	80.77	80.66	74.59	74.35
23	50(0)	60(0)	60(0)	20(−2)	4.80	4.71	83.88	82.97	70.73	70.83
24	50(0)	60(0)	60(0)	60(2)	6.23	6.28	87.04	87.35	75.09	75.41
25	50(0)	60(0)	60(0)	40(0)	6.06	6.15	85.52	86.07	80.03	70.83
26	50(0)	60(0)	60(0)	40(0)	6.28	6.15	86.47	86.07	79.14	75.41
27	50(0)	60(0)	60(0)	40(0)	6.18	6.15	85.76	86.07	79.95	79.73
28	50(0)	60(0)	60(0)	40(0)	6.11	6.15	86.13	86.07	80.13	79.73
29	50(0)	60(0)	60(0)	40(0)	6.16	6.15	86.23	86.07	79.23	79.73
30	50(0)	60(0)	60(0)	40(0)	6.10	6.15	86.32	86.07	79.90	79.73

**^a^** Rls is ratio of liquid to solid (mL/g); **^b^** Et is extraction temperature (°C); **^c^** T is extraction time (min); **^d^** Ec is ethanol concentration (%, *v*/*v*); exp: experimental; pre: predicted.

Multiple regression analysis was applied on the experiment data, and the software generated three regression equations which demonstrated the empirical relationship between the response variables and the test variables (Table 2), where Y is the response values of TFC, ABTS, and DPPH; X_1_, X_2_, X_3_, and X_4_ are the coded values of the ratio of solid to liquid, extraction temperature, extraction time, and ethanol concentration, respectively.

**Table 2:** Regression models fitted to the experimental data of response variables.

	Response	Model Equation ^a^
Y_1_	TFC(mg RE/g)	Y_1_ = 6.15 + 0.18X_1_ + 0.034X_2_ − 0.027X_3_ + 0.39X_4_ − 0.053X_1_X_2_ − 0.090X_1_X_3_ − 0.001724X_1_X_4_ − 0.066X_2_X_3_ + 0.052X_2_X_4_ + 0.040X_3_X_4_ − 0.13X_1_^2^ − 0.15X_2_^2^ − 0.2X_3_^2^ − 0.16X_4_^2^
Y_2_	ABTS radical-scavenging capability (%)	Y_2_ = 225.29 + 11.75X_1_ + 4.41X_2_ + 16.40X_3_ + 28.77X_4_ + 0.64X_1_X_2_ + 8.87X_1_X_3_ + 4.80X_1_X_4_ + 0.38X_2_X_3_ + 0.031X_2_X_4_ + 16.73X_3_X_4_ + 57.38X_1_^2^ + 19.46X_2_^2^ + 85.62X_3_^2^ + 1.42X_4_^2^
Y_3_	DPPH radical-scavenging capability (%)	Y_3_ = 277.99 + 17.73X_1_ + 14.15X_2_ + 3.10X_3_ + 31.50X_4_ + 43.73X_1_X_2_ + 13.82X_1_X_3_ + 3.36X_1_X_4_ + 0.23X_2_X_3_ + 3.79X_2_X_4_ + 3.21X_3_X_4_ + 48.28X_1_^2^ + 41.95X_2_^2^ + 37.23X_3_^2^ + 74.86X_4_^2^

**^a^** X_1_: Ratio of liquid to solid (mL/g); X_2_: Extraction temperature (°C); X_3_: Extraction time (min); X_4_: Ethanol concentration (%, *v*/*v*).

The ANOVA results, goodness-of-fit and the adequacy of the fitted models are summarized in Table 3 and Table 4. For any terms in the model, a large regression coefficient and a small *p*-value indicate a more significant effect of that term on the respective response variable [47].

**Table 3:** Analysis of variance results for the multiple regression to predict flavonoids.

Source		Sum of Squares	Degree of Freedom	Mean Square	*f*-Value	*p*-Value	Significant ^b^
Y_1_	Model ^a^	6.93	14	0.50	69.03	˂0.0001	**
	X_1_	0.82	1	0.82	115.29	˂0.0001	**
	X_2_	0.029	1	0.029	4.02	0.0634	
	X_3_	0.018	1	0.018	2.55	0.1309	
	X_4_	3.7	1	3.70	522.30	˂0.0001	**
	X_1_X_2_	0.045	1	0.045	6.37	0.0234	*
	X_1_X_3_	0.13	1	0.13	18.20	0.0007	**
	X_1_X_4_	4.758 × 10^−5^	1	4.758 × 10^−5^	6.708 × 10^−3^	0.9358	
	X_2_X_3_	0.069	1	0.069	9.79	0.0069	**
	X_2_X_4_	0.044	1	0.044	6.15	0.0255	*
	X_3_X_4_	0.026	1	0.026	3.66	0.0749	
	X_1_^2^	0.45	1	0.45	63.11	˂0.0001	**
	X_2_^2^	0.61	1	0.61	85.71	˂0.0001	**
	X_3_^2^	1.09	1	1.09	153.67	˂0.0001	**
	X_4_^2^	0.74	1	0.74	103.82	˂0.0001	**
	Residual	0.11	15	7.092× 10^−3^			
	Lack of fit	0.076	10	7.552× 10^−3^	1.22	0.4360	
	Pure error	0.031	5	6.172× 10^−3^			
	R^2^ = 0.9849; Adj R^2^ = 0.9708; Pred R^2^ = 0.9319; Adeq Precision = 26.390; C.V.% = 1.49						

^a^ X_1_: Ratio of liquid to solid (mL/g); X_2_: Extraction temperature (°C); X_3_: Extraction time (min); X_4_: Ethanol concentration (%, *v*/*v*). ^b^ **: indicate highly significant (*p* ˂ 0.01), *: indicate significant (*p* ˂ 0.05).

**Table 4:** Analysis of variance results for the multiple regressions to predict ABTS and DPPH radical scavenging capacity of extracts.

Source	Degree of Freedom	ABTS Radical Scavenging Capacity (%)					DPPH Radical Scavenging Capacity (%)						
		Sum of Squares	Mean Square	*f*-Value	*p*-Value	Significant ^b^	Sum of Squares	Mean Square	*f*-Value	*p*-Value		Significant ^b^	
Y_2_ Model ^a^	14	225.29	16.09	54.25	˂0.0001	**	Y_3_ 277.99	19.86	44.25	˂0.0001		**	
X_1_	1	11.75	11.75	39.62	˂0.0001	**	17.73	17.73	39.51	˂0.0001		**	
X_2_	1	4.41	4.41	14.86	0.0016	*	14.15	14.15	31.53	˂0.0001		**	
X_3_	1	16.40	16.40	55.29	˂0.0001	**	3.10	3.10	6.90	0.0190		*	
X_4_	1	28.77	28.77	97.01	˂0.0001	**	31.50	31.50	70.21	˂0.0001		**	
X_1_X_2_	1	0.64	0.64	2.16	0.1626		43.73	43.73	97.46	0.0234		*	
X_1_X_3_	1	8.87	8.87	29.91	˂0.0001	**	13.82	13.82	30.80	0.0007		**	
X_1_X_4_	1	4.80	4.80	16.19	0.0011	**	3.36	3.36	7.48	0.9358			
X_2_X_3_	1	0.38	0.38	1.29	0.2732		0.23	0.23	0.50	0.0069		**	
X_2_X_4_	1	0.031	0.031	0.10	0.7508		3.79	3.79	8.44	0.0255		*	
X_3_X_4_	1	16.73	16.73	56.40	˂0.0001	**	3.21	3.21	7.15	0.0749			
X_1_^2^	1	57.38	57.38	193.46	˂0.0001	**	48.28	48.28	107.60	˂0.0001		**	
X_2_^2^	1	19.46	19.46	65.60	˂0.0001	**	41.95	41.95	93.49	˂0.0001		**	
X_3_^2^	1	85.62	85.62	288.68	˂0.0001	**	37.23	37.23	82.98	˂0.0001		**	
X_4_^2^	1	1.42	1.42	4.78	0.0451	*	74.86	74.86	166.84	˂0.0001		**	
Residual	15	4.45	0.30				6.73	0.45					
Lack of fit	10	3.79	0.38	2.87	0.1279		5.81	0.58	3.17	0.4527			
Pure error	5	0.66	0.13				0.92	0.18					
	R^2^ = 0.9806; Adj R^2^ = 0.9626; Pred R^2^ = 0.9009; Adeq Precision = 25.970; C.V.% = 0.66						R^2^ = 0.9764; Adj R^2^ = 0.9543; Pred R^2^ = 0.8788; Adeq Precision = 23.316; C.V.% = 0.89						

^a^ X_1_: Ratio of liquid to solid (mL/g); X_2_: Extraction temperature (°C); X_3_: Extraction time (min); X_4_: Ethanol concentration (%, *v*/*v*). ^b^ **: indicate highly significant (*p* ˂ 0.01), *: indicate significant (*p* ˂ 0.05).

ANOVA for the quadratic regression models indicated that the three models were very significant (*p* ˂ 0.01). The quality of fit of the model is usually evaluated by the determination coefficient (R^2^), predicted determination coefficient (R^2^_pred_), and adjusted determination coefficient (R^2^_adj_). The values of the determination coefficient (R^2^ =0.9849, 0.9806, and 0.9764 for TFC, ABTS, and DPPH radical-scavenging activity, respectively) of the quadratic regression model indicated that only 1.61%, 1.94%, and 2.36% of the total variations could not be explained by the model, respectively. The adjusted determination coefficient (R^2^_adj_ = 0.9708, 0.9626, 0.9543) showed the high degree of correlation between the experimental and predicted values. The models also showed statistically-insignificant lack of fit, as is evident from the computed *f*-values of 0.4360, 0.1279, and 0.4527 for TFC, ABTS and DPPH methods, respectively. Based on the factors above, it was deduced that the model was appropriate to predict the responses.

For the extraction yield of TFC and antioxidants assayed by ABTS and DPPH methods, each response can be assigned a significance degree relative to the other responses. The results indicated that all the quadratic terms and liner terms as the ratio of liquid and solid (X_1_) and ethanol concentration (X_4_) and the interaction between X_1_X_3_, X_2_X_3_, were highly significant (*p* ˂ 0.01) on the total flavonoids yield (Y_1_), whereas the linear and quadratic effects of all independent variables were found to be significant (*p* ˂ 0.05) on ABTS (Y_2_) and DPPH (Y_3_) radical-scavenging capacity of extracts from alfalfa (Table 3). Moreover, three interactions of X_1_X_3_, X_1_X_4_, and X_3_X_4_ were significant (*p* ˂ 0.01) on ABTS radical-scavenging capacity, while only two interactions of X_1_X_3_, X_2_X_3_ were significant (*p* ˂ 0.01) on DPPH radical-scavenging capacity.

#### 2.2.2. Analysis of Response Surfaces

Three-dimensional response surface plots drawn by Design Expert Software (Version 8.0.5, Stat-Ease Inc., Minneapolis, MN, USA) were constructed to investigate the main and the interactive effects on the recoveries of TFC and scavenging capacities of ABTS and DPPH radicals. The surface plots were generated by holding two variables at zero level, while varying the other two variables within the experiment range under investigation.

**Figure 5:**
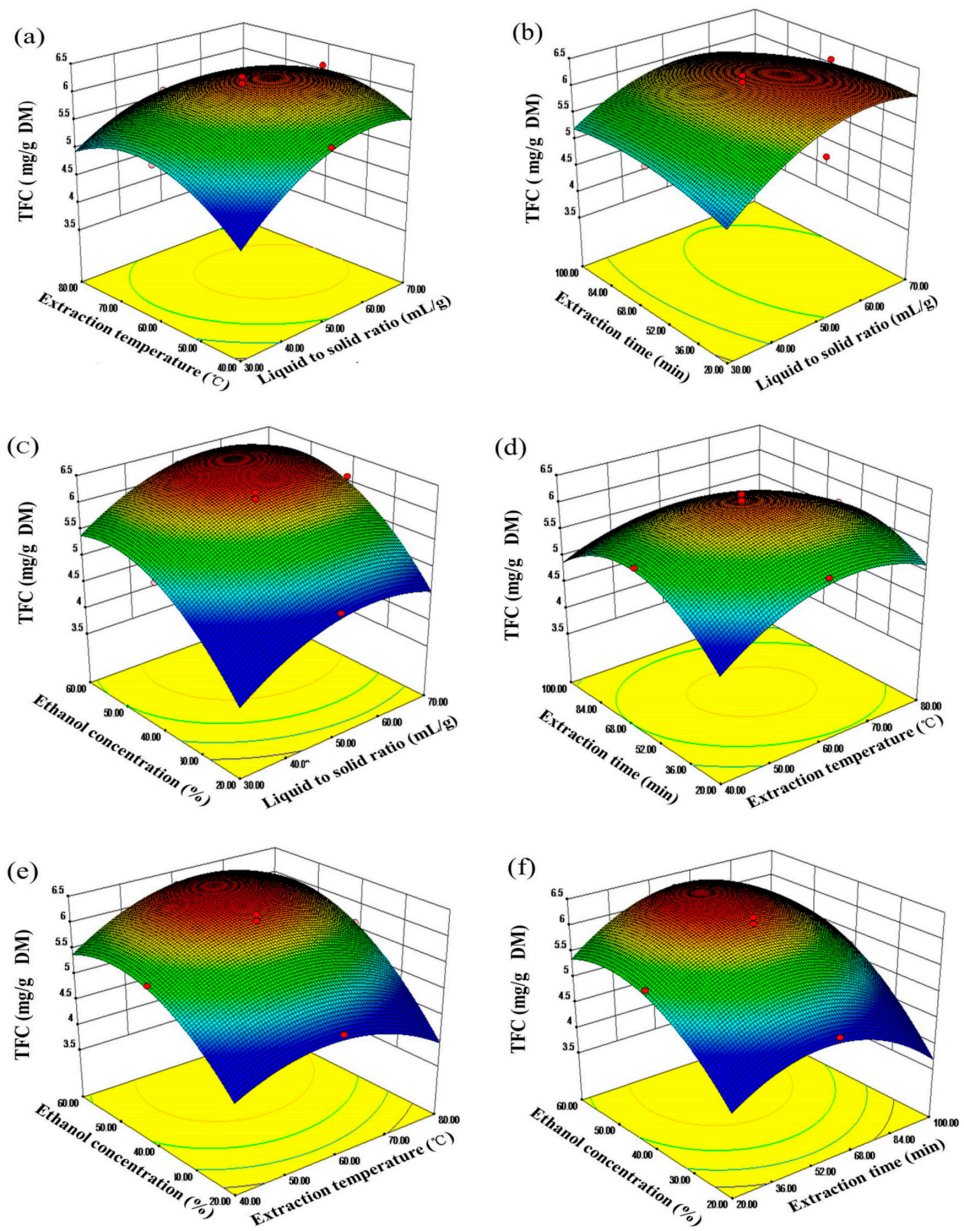
Response surface plots (3D) showing the effects of variables on total flavonoid extraction. (**a**) liquid to solid ratio and temperature; (**b**) liquid to solid ratio and time; (**c**) liquid to solid ratio and Ethanol concentration; (**d**) time and temperature; (**e**) temperature and ethanol concentration; (**f**) time and ethanol concentration.

**Figure 6:**
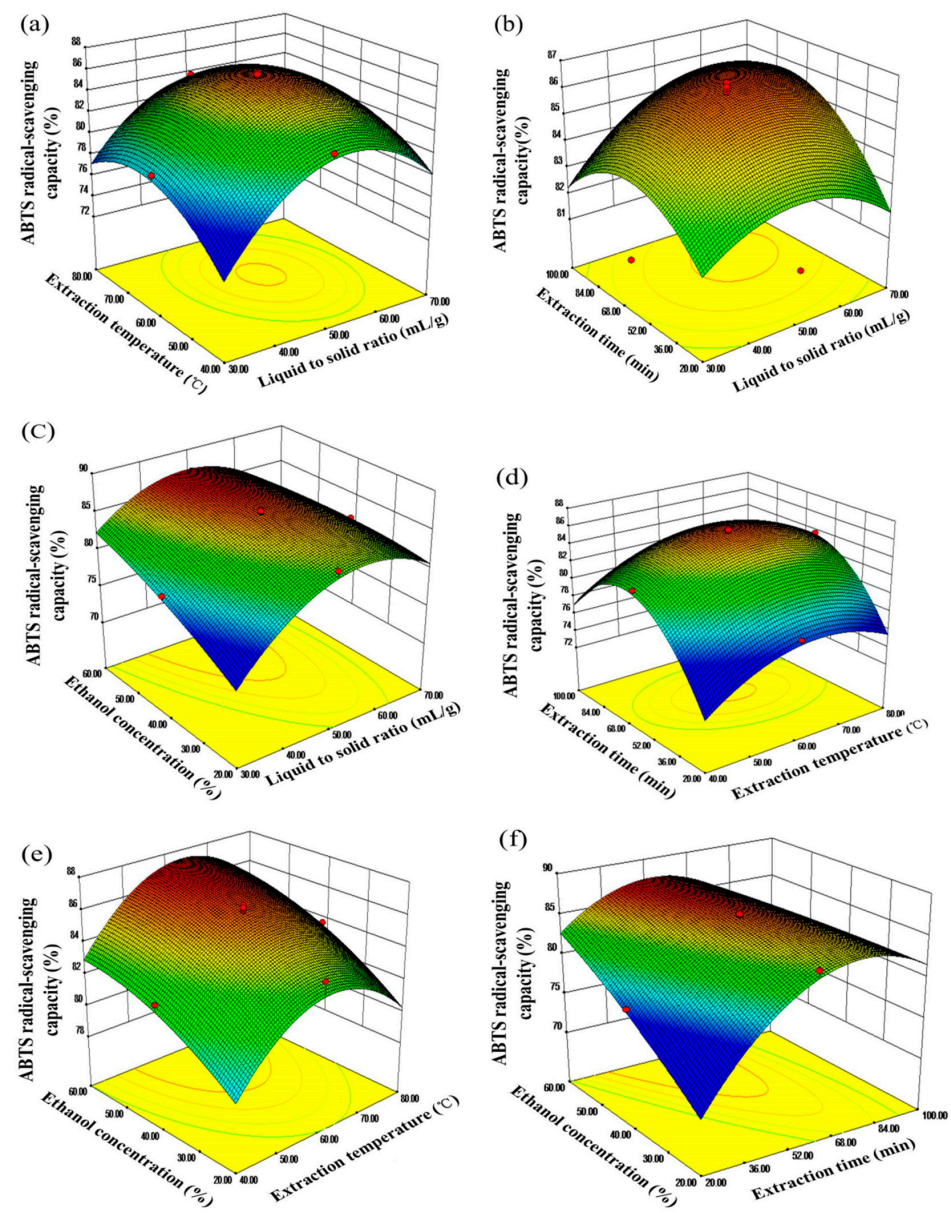
Response surface plots (3D) showing the effects of the extraction parameters on ABTS radical-scavenging capacity of alfalfa extracts. (**a**) liquid to solid ratio and temperature; (**b**) liquid to solid ratio and time; (**c**) liquid to solid ratio and Ethanol concentration; (**d**) time and temperature; (**e**) temperature and ethanol concentration; (**f**) time and ethanol concentration.

**Figure 7:**
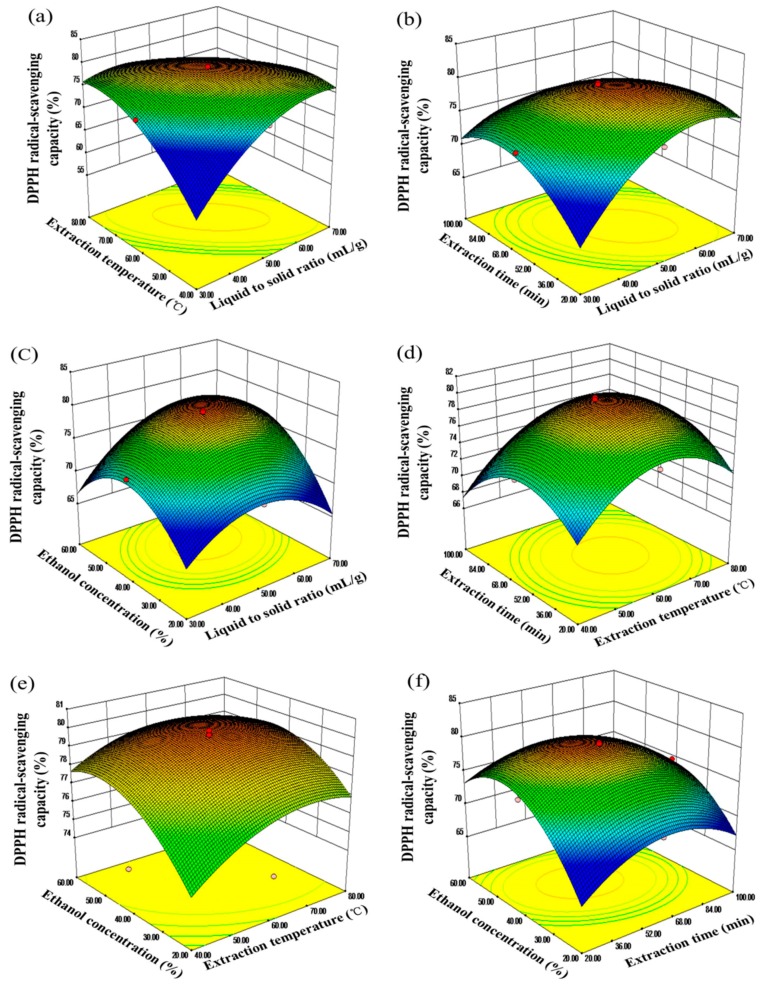
Response surface plots (3D) showing the effects of the extraction parameters on DPPH radical-scavenging capacity of alfalfa extracts. (**a**) liquid to solid ratio and temperature; (**b**) liquid to solid ratio and time; (**c**) liquid to solid ratio and Ethanol concentration; (**d**) time and temperature; (**e**) temperature and ethanol concentration; (**f**) time and ethanol concentration.

The effects of the liquid to solid ratio interaction with the other factors on the recovery of TFC, ABTS, and DPPH radical-scavenging antioxidants are shown in Figure 5a–c, Figure 6a–c, and Figure 7a–c. It was obvious that the lower recovery in the lower solvent volume, and extraction recoveries increased with an increase the ratio from 30:1 (mL/g) to 50:1 (mL/g), but the ratio over 60:1 (mL/g) appeared to diminish extraction yield.

For the extraction temperature, as can be seen from Figure 5a,d,e, a quadratic effect was detected for all response variables, and the extraction yield of flavonoids increased from 40 to 60 °C. This might be due to the increase in temperature accelerating the mass transfer. Similar results have also been reported for the extraction of flavonoids from other materials [48,49]. Figure 6a,d,e and Figure 7a,d,e shows the effects of extraction temperature with each of the three other factors on the recovery of ABTS and DPPH radical-scavenging antioxidants. In all situations, scavenging capacity of ABTS and DPPH radicals increased with increasing extraction temperature from 40°C to 55 °C, while more than 60 °C appeared to be disadvantaged on the extraction of antioxidants.

In Figure 6, when the response surface plots were developed for the extraction time interaction with the other three factors, it can be seen that the maximum extraction yield could be achieved when the extraction time around 60 min. As shown in Figure 6b,d,f and Figure 7b,d,f, it indicated that the maximum scavenging capacity of ABTS and DPPH radicals appeared when the extraction time reached 60 min and 45 min, respectively.

As expected, ethanol concentration exerted a quadratic effect on the recovery of TFC, ABTS, and DPPH radical-scavenging capacity antioxidants. The yield of flavonoids increased to the highest value with an ethanol concentration around 50% (Figure 5c,e,f). Likewise, a greater value of the scavenging capacity of DPPH radicals was obtained when the ethanol concentration increased from 20% to 46% (Figure 7c,e,f), and decreased thereafter. However, the capacity of ABTS radical-scavenging decreased when the concentration was above 60% (Figure 6c,e,f).

#### 2.2.3. Verification of Predictive Model

The optimum UAE conditions for the total flavonoids and the antioxidants from alfalfa are given in Table 5. The ratio of liquid to solid in the range 47.29–60.30 mL/g, extraction temperature in the range of 54.56–63.73 °C, extraction time of 45.59–57.08 min, and ethanol concentration in the range of 46.67%–60% produced the optimal TFC (6.46 mg RE/g dried plant) and total antioxidant activities (87.76% for ABTS and 80.48% for DPPH) from alfalfa. The predicted results matched well with the experimental values (Table 5) obtained using optimum extraction conditions, which were confirmed with the CV ranged from 0.63% to 1.32%.

**Table 5:** Experimental and predicted values of response variables on optimal conditions.

Response	Optimum Extraction Conditions ^b^				Maximum Value		% Difference (CV)
	X_1_	X_2_	X_3_	X_4_	Experimental ^a^	Predicted	
Y_1_(RE mg/g)	57.16	62.33	57.08	52.14	6.40 ± 0.08	6.46	1.32
Y_2_ (%)	47.29	63.73	51.62	60	87.38 ± 0.55	87.76	0.63
Y_3_ (%)	60.30	54.56	45.59	46.67	80.72 ± 0.54	80.48	0.67

**^a^** Results are means ± SD (*n* = 3); **^b^** X_1_: Ratio of liquid to solid (mL/g); X_2_: Extraction temperature (°C); X_3_: Extraction time (min); X_4_: Ethanol concentration (%, *v*/*v*).

#### 2.2.4. Comparison with Conventional Extraction

In order to compare extraction efficiency, UAE, which was performed at the optimum conditions by RSM, was applied to compare with conventional method. TFC, total phenolic content (TPC), and antioxidant activity of extracts obtained from alfalfa using UAE and conventional extraction methods are presented in Table 6. Compared with the conventional extraction method, TFC and TPC of the crude extracts by UAE were higher than that by conventional extraction method. The results indicated that TFC was highest in E_1_ (39.15 mg RE/g) among the four samples. However, the TPC determined by Folin-Ciocalteau procedure in E_1_ (66.51 mg GAE/g) and E_2_ (68.02 mg GAE/g) was higher than E_3_ (58.35 mg GAE/g) and E_4_ (48.36 mg GAE/g), and there were no significant differences between E_1_ and E_2_. The result was supported by the results of [50], who stated that 60% (*v*/*v*) ethanol provided the highest total phenolic extraction.

**Table 6:** The content of total flavonoids, phenolic, and antioxidant capacity of extracts prepared under three optimal UAE conditions and conventional methods.

Methods		TFC	TPC	ABTS	DPPH	FRAP
Ultrasonic-assisted extraction	E_1_	39.15 ± 0.63 ^a^	66.51 ± 1.52 ^a^	86.55 ± 0.07 ^a,b^	79.23 ± 0.68 ^a^	33.18 ± 0.14 ^b^
	E_2_	36.13 ± 0.68 ^b^	68.02 ± 0.43 ^a^	87.38 ± 0.68 ^a^	71.98 ± 0.13 ^b^	36.24 ± 0.11 ^a^
	E_3_	34.88 ± 0.35 ^b^	58.35 ± 0.11 ^b^	85.58 ± 0.54 ^b^	80.72 ± 0.67 ^a^	32.68 ± 0.28 ^c^
Conventional	E_4_	29.73 ± 1.20 ^c^	48.36 ± 0.29 ^c^	70.85 ± 0.68 ^c^	66.73 ± 3.66 ^c^	32.65 ± 0.19 ^c^

Results are means ± SD (*n* = 3). Values followed by different letters are significantly different at *p* < 0.05. Sample E_1_, E_2_, E_3_ and E_4_ are the dried crude power extracted under the optimal conditions of Y_1_, Y_2_, Y_3_ and conventional method, respectively; TFC: total flavonoid content (mg RE/g), TPC: total phenolic content (mg GAE/g), ABTS: ABTS radical-scavenging ability (%), DPPH: DPPH radical- scavenging capacity (%); FRAP: ferric-reducing Antioxidant power (mg AA/g).

Meanwhile, it can be seen that UAE demonstrated a positive influence on antioxidant extraction than the conventional method. The antioxidant activity of extracts power was determined using ABTS, DPPH radical scavenging and FRAP assay. The antioxidant capacities determined by ABTS assay were 86.55%, 87.38%, 85.58%, and 70.85%, respectively, and the values of E_1_, E_2_, and E_3_ which extracted with UAE is higher than that of E_4_. Using DPPH assay, the antioxidant activities of E_1_ and E_3_ is significantly higher than E_2_ and E_4_. The antioxidant capacity assayed by FRAP was 33.18 mg AA/g, 36.24 mg AA/g, 32.68 mg AA/g, and 32.65 mg AA/g for the four samples, respectively, and value in E_2_ is higher than the other three samples. The high efficiency of UAE found in the present study might result from strong surface damage on alfalfa so that the flavonoid and antioxidant compounds dissolved more easily in the solvent. Hence, UAE is a promising extraction technique for flavonoid extraction, which has a high efficiency and reduced the extraction time, compared to conventional extraction.

## 3. Materials and Methods

### 3.1. Sample Collection and Pretreatment

The fresh whole alfalfa was collected at full bloom from a local farm. The whole alfalfa were collected and washed thoroughly in potable water, and then oven dried for 24 h at 65 °C. Dried material were then powdered and subsequently sieved through a 0.45 mm sifter (40 mesh). The obtained powder was stored in plastic sealed bags and kept in a dry environment prior to use. Rutin, DPPH, Folin-Ciocalteu’s phenol reagent, and ABTS were purchased from Sigma Chemicals Co. (St. Louis, MO, USA). All other chemicals used in this experiment were of analytical grade and purchased from Beijing Chemical Co. (Beijing, China). All the stock solutions were prepared by purified deionized water (MilliQ purification system, Millipore, Molsheim, France).

### 3.2. Extraction of Flavonoids and Antioxidants

#### 3.2.1. Ultrasound-Assisted Extraction of Flavonoids and Antioxidants from Alfalfa

Ultrasonic-assisted extraction was performed in a digitally-controlled ultrasonic bath (KQ3200E, Kunshan Ultrasonic Instrument Co. Jiangsu, China). The extraction variables were selected according to [51]. Sample of 3 g was placed into a flask (250 mL), soaked with ethanol solvent at the given concentration in a scheduled ratio of liquid to solid and then placed in an ultrasonic cleaning bath at 40 kHz for a certain time at a constant temperature. Extracts were filtered through a filter paper under vacuum and the residue was extracted again (three times) with the same volume of fresh solvent. Then, filtrates were combined and concentrated using a rotary evaporator at 50 °C under vacuum. Finally, the filtrate was prepared to a constant volume (150 mL) using 60% ethanol for estimation of flavonoids and antioxidant measurements through various chemical assays.

#### 3.2.2. Conventional Extraction

Heat reflux extraction is the conventional extraction process in Chinese pharmacopoeia. Sample of 3 g was soaked with 70% ethanol solvent (1:25 solid to liquid ratio, g/mL) and extracted by refluxing at 80 °C. Extracts were then filtered through a filter paper under vacuum. The filtrate was collected and the residue was extracted again (three times) with the same volume of fresh solvent. Then, filtrates were combined and concentrated to a constant volume (150 mL) for estimation of flavonoids and antioxidant measurements through various chemical assays.

### 3.3. Single-Factor Experiments

To investigate the extraction of flavonoids and antioxidant compounds under different conditions, single-factor tests were employed first to determine the optimal extraction parameters (ratio of liquid to solid, extraction temperature, extraction time, and ethanol concentration). Ratio of liquid to solid: each material (3 g of power filtered through 40 mesh) was dissolved in different volumes of 40% ethanol (60, 90, 120, 150, 180, and 210 mL). The extraction temperature was 60 °C, extraction time 60 min. Extraction temperature: Each material (3 g of dried power filtered through 40 mesh) was mixed with 40% ethanol 150 mL (based on the results from the above step). The extraction temperatures were 30, 40, 50, 60, 70, and 80 °C and extraction time 60 min. Extraction time: Each material (3 g of dried power filtered through 40 mesh) was mixed with 40% ethanol 150 mL at 60 °C (based on the results from the above step) and the extraction times were 20, 40, 60, 80, 100, and 120 min, respectively. Ethanol concentration: Each material (3 g of dried power filtered through 40 mesh) was mixed with 150 mL different ethanol solvents 20%, 30%, 40%,50%, 60%, and 70% (*v*/*v*). The extraction temperature was 60 °C and extraction time 60 min (based on the results from the above step). All the experiments were conducted in triplicate.

### 3.4. Experimental Design

A four independent variable, five-level central composite rotatable response surface design (CCRD) (Design Expert Software, Version 8.0.5, Stat-Ease Inc., Minneapolis, MN, USA) was used for the optimization of the extraction conditions. Four selected independent variables were the ratio of liquid to solid (mL/g, X_1_), extraction temperature (°C, X_2_), extraction time (min, X_3_), and ethanol concentration (*v*/*v*, X_4_). The levels for each independent variable were chosen based on three responses of the crude extracts: the total flavonoids as a priority, followed by ABTS radical-scavenging capacity, and DPPH radical-scavenging capacity. The coded and uncoded (actual) levels of the independent variables are shown in Table 1. A second-order polynomial regression model was used to express the yield values of TFC, DPPH, and ABTS radical-scavenging capabilities as a function of the independent variables:

(1) $${\text{Y=β}}_{\text{0}}\text{+}\overset{\text{4}}{\underset{\text{j = 1}}{\sum }}{\text{β}}_{\text{j}}{\text{x}}_{\text{j}}\text{+}\overset{\text{4}}{\underset{\text{j = 1}}{\sum }}{\text{β}}_{\text{jj}}{\text{x}}_{\text{j}}^{\text{2}}\text{+}\overset{\text{3}}{\underset{\text{i = 1}}{\sum }}\overset{\text{4}}{\underset{\text{j = i + 1}}{\sum }}{\text{β}}_{\text{ij}}{\text{X}}_{\text{i}}{\text{X}}_{\text{j}}$$

where Y represents the response; β_0_ is a constant that represents the model intercept coefficient; β_j_, β_jj_, and β_ij_ are the linear, quadratic, and interactive coefficients, respectively; X_i_ and X_j_ represents the coded independent variables, respectively.

### 3.5. Determination of Total Flavonoid Content

The total flavonoid content from alfalfa in extracts was performed according to the method in [52] with some modifications. The reaction mixture contained 5.0 mL of extract, 5 mL of 60% ethanol, and 1 mL of 5% sodium nitrite. Six minutes later, 1 mL of 10% aluminum nitrite was added. In the next six minutes, 10 mL of 1 M sodium hydroxide solution were added and the volume was increased to 25 mL by adding 60% ethanol. Immediately, the reaction mixture absorbance was measured by a spectrophotometer at 510 nm against a blank (control) and used to calculate TFC using rutin as a standard. Measurements were calibrated to a standard curve of prepared rutin solution ranging from 0–0.048mg/mL with y = 12.219x + 0.0036, (R^2^ = 0.9998). TFC is calculated as follows:

(2) $$\text{TFC = }\frac{\text{the flavonoid content of extracts }\left(\text{mg RE}\right)}{\text{weight of alfalfa powder (g) }}$$

### 3.6. Determination of Total Phenolic Content

Total phenolic content from extracts was measured according to the Folin-Ciocalteu procedure, as described by [53]. The extract was measured at absorbance of 756 nm. Measurements were calibrated to a standard curve of prepared gallic acid solution ranging from 0–0.2 mg/mL with y = 4.214x − 0.0118, (R^2^ = 0.999) and the results was then expressed as mg of gallic acid equivalents (GAE) per g of dry crude extracts.

(3) $$\text{TPC = }\frac{\text{the phenolic content of extracts (mg GAE)}}{\text{weight of dried extracts (g)}}$$

### 3.7. Determination of Antioxidant Activity

#### 3.7.1. DPPH Radical Scavenging Capacity Measurement

The radical-scavenging ability of DPPH was measured according to the method of [54]. Thus, the DPPH radical solution was produced by gently mixing 0.1 mM DPPH solution (0.75 mL) and the sample solution (1.5 mL) with various concentrations. This was allowed to stand in the dark for 30 min, and absorbance was measured at 517 nm. The free radical scavenging activity was calculated as following:

(4) $$\text{ Scavenging effect }\left(\text{\%}\right)\text{ = }\left[\text{1 }-\;\frac{{\text{A}}_{\text{1}}\;-{\text{ A}}_{\text{2}}}{{\text{A}}_{\text{0}}}\right]\text{ × 100\%}$$

where A_1_ was the absorbance of the sample, A_0_ was the absorbance of the solvent control, and A_2_ was the absorbance of the reagent blank without DPPH.

#### 3.7.2. ABTS Radical Scavenging Capacity Measurement

The radical-scavenging ability of ABTS was estimated by a method according to [55] with some modifications. The ABTS radical solution was produced by adding 100 mL of 7 mM ABTS solution to 100 mL of 2.45 mM potassium persulfate solution. It was allowed to stand in the dark at room temperature for 12–16 h. The ABTS radical solution was adjusted to an absorbance of 0.7 ± 0.02 with ethanol at 734 nm before usage. Thus, 3.9 mL ABTS radical solution was added with the sample solution or ethanol and the mixture was allowed to stand at 30 °C to obtain a stable absorbance. Then it was measured at 734 nm against a blank. The radical scavenging capacity was calculated as follows:

(5) $$\text{ Scavenging effect }\left(\text{\%}\right)\text{ = }\left[\frac{{\text{A}}_{\text{blank}}\;-{\text{ A}}_{\text{sample}}}{{\text{A}}_{\text{blank}}}\right]\text{×100\% }$$

where A_blank_ was the absorbance of all the reagents without the sample, and the A_sample_ was the absorbance of the sample.

#### 3.7.3. Ferric-Reducing Antioxidant Power Assay (FRAP)

The FARP was measured according to [56] with some modifications. A fresh working solution was prepared by mixing 100 mL 300 mmol/L acetate buffer (pH = 3.6), 10 mL 10 mmol/L TPTZ solution in 40 mmol/L HCl, and 10 mL 20 mmol/L FeCl_3_ solution. The reaction was carried out by adding sample (20 μL) to the FRAP solution (180 μL) for 10 min at 37 °C, and absorbance at 593 nm was recorded. The control assay was performed using 180 μL of FRAP reagent and 20 μL of ethanol. The calibration curve was prepared with ascorbic acid (AA). The results were expressed as ascorbic acid equivalent produced in the samples.

### 3.8. Statistical Analysis

All results were centered at using three parallel experiments. Results were expressed as mean ± standard deviation and analyzed by Statistical Analysis System 9.2 (SAS Institute Inc., Cary, NC, USA). Analysis of variance was performed by ANOVA procedure. *p*-value of 0.05 or 0.01 was considered to be statistically significant.

## 4. Conclusions

Based on the single-factor experiments, RSM was used to optimize the extraction variables. The second-order model developed for the yield of flavonoids from alfalfa was extremely significant. The optimum extraction parameters were shown in Table 5. In addition, the results obtained from the study provided that ultrasonic-assisted technique could be used as an effective method to extract flavonoids from alfalfa and the extracts of alfalfa possess ABTS and DPPH radical-scavenging abilities. This study provides constructive information to further investigate alfalfa for its antioxidant properties and application possibilities.

## References

[1] Karimi E., Oskoueian E., Oskoueian A., Omidvar V., Hendra R., Nazeran H. (2013). Insight into the functional and medicinal properties of Medicago sativa (Alfalfa) leaves extract. J. Med. Plant Res..

[2] Bora K.S., Sharma A. (2011). Phytochemical and pharmacological potential of Medicago sativa: A review. Pharm. Biol..

[3] Gaweł E. (2012). Chemical compositions of lucerne leaf extract (EFL) and its applications as a phytobiotic in human nutrition. Acta Sci. Pol. Technol. Aliment..

[4] Zhu J.M., Li N., Zhang Y.J., Li X.D., Wang C.Z. (2009). The research progress of alfalfa flavonoids. Pratac. Sci..

[5] Wang S.P., Dong X.F., Ma H., Cui Y.M., Tong J.M. (2014). Purification, characterisation and protective effects of polysaccharides from alfalfa on hepatocytes. Carbohydr. Polym..

[6] Sun Y., Long R.C., Zhang T.J., Yang Q.C., Zhou H. (2013). Advances in the study of alfalfa saponin. Cao Ye Xue Bao.

[7] Pietta P.G. (2000). Flavonoids as antioxidants. J. Nat. Prod..

[8] Heim K.E., Tagliaferro A.R., Bobilya D.J. (2002). Flavonoid antioxidants: chemistry, metabolism and structure-activity relationships. J. Nutr. Biochem..

[9] Caunii A., Pribac G., Grozea I., Gaitin D., Samfira I. (2012). Design of optimal solvent for extraction of bio-active ingredients from six varieties of Medicago sativa. Chem. Cent. J..

[10] Stochmal A., Piacente S., Pizza C., de Riccardis F., Leitz R., Oleszek W. (2001). Alfalfa (*Medicago sativa* L.) flavonoids. 1. Apigenin and luteolin glycosides from aerial parts. J. Agric. Food Chem..

[11] Stochmal A., Simonet A.M., Macias F.A., Oleszek W. (2001). Alfalfa (*Medicago sativa* L.) flavonoids. 2. Tricin and chrysoeriol glycosides from aerial parts. J. Agric. Food Chem..

[12] Wang X., Wu Q., Wu Y., Chen G., Yue W., Liang Q. (2012). Response surface optimized ultrasonic-assisted extraction of flavonoids from Sparganii rhizoma and evaluation of their *in vitro* antioxidant activities. Molecules.

[13] Gao Z., Huang K., Yang X., Xu H. (1999). Free radical scavenging and antioxidant activities of flavonoids extracted from the radix of Scutellaria baicalensis Georgi. Biochim. Biophys. Acta.

[14] Zhang Y.M., Yan S.J., Cao Z.Z., Shi S.L. (2008). Methodological Study for Total Flavonoid Extraction from Alfalfa by Microwave Assistance. Acta Agrestia Sin..

[15] Wang L., Yang B., Du X., Yi C. (2008). Optimisation of supercritical fluid extraction of flavonoids from Pueraria lobata. Food Chem..

[16] Huang W., Xue A., Niu H., Jia Z., Wang J. (2009). Optimised ultrasonic-assisted extraction of flavonoids from *Folium eucommiae* and evaluation of antioxidant activity in multi-test systems *in vitro*. Food Chem..

[17] Novak I., Janeiro P., Seruga M., Oliveira-Brett A.M. (2008). Ultrasound extracted flavonoids from four varieties of Portuguese red grape skins determined by reverse-phase high-performance liquid chromatography with electrochemical detection. Anal. Chim. Acta.

[18] Wang J., Sun B., Cao Y., Tian Y., Li X. (2008). Optimisation of ultrasound-assisted extraction of phenolic compounds from wheat bran. Food Chem..

[19] Wang L., Weller C.L. (2006). Recent advances in extraction of nutraceuticals from plants. Trends Food Sci. Technol..

[20] Bezerra M.A., Santelli R.E., Oliveira E.P., Villar L.S., Escaleira L.A. (2008). Response surface methodology (RSM) as a tool for optimization in analytical chemistry. Talanta.

[21] Ji Y.B., Dong F., Ma D.B., Miao J., Jin L.N., Liu Z.F., Zhang L.W. (2012). Optimizing the extraction of anti-tumor polysaccharides from the fruit of *Capparis spionosa* L. by response surface methodology. Molecules.

[22] Lai J., Xin C., Zhao Y., Feng B., He C., Dong Y., Fang Y., Wei S. (2013). Optimization of ultrasonic assisted extraction of antioxidants from black soybean (*Glycine max* var.) sprouts using response surface methodology. Molecules.

[23] Myers R.H., Montgomery D.C., Anderson-Cook C.M. (2009). Response Surface Methodology: Process and Product Optimization Using Designed Experiments.

[24] Dahmoune F., Nayak B., Moussi K., Remini H., Madani K. (2015). Optimization of microwave-assisted extraction of polyphenols from *Myrtus communis* L. leaves. Food Chem..

[25] Lai J., Wang H., Wang D., Fang F., Wang F., Wu T. (2014). Ultrasonic extraction of antioxidants from Chinese sumac (*Rhus typhina* L.) fruit using response surface methodology and their characterization. Molecules.

[26] Zhang G., He L., Hu M. (2011). Optimized ultrasonic-assisted extraction of flavonoids from *Prunella vulgaris* L. and evaluation of antioxidant activities *in vitro*. Innov. Food Sci. Emerg. Technol..

[27] Yang L., Cao Y.L., Jiang J.G., Lin Q.S., Chen J., Zhu L. (2010). Response surface optimization of ultrasound-assisted flavonoids extraction from the flower of *Citrus aurantium* L. var. *amara* Engl.. J. Sep. Sci..

[28] Zou T.B., Xia E.Q., He T.P., Huang M.Y., Jia Q., Li H.W. (2014). Ultrasound-assisted extraction of mangiferin from mango (*Mangifera indica* L.) leaves using response surface methodology. Molecules.

[29] Pompeu D., Silva E., Rogez H. (2009). Optimisation of the solvent extraction of phenolic antioxidants from fruits of Euterpe oleracea using Response Surface Methodology. Bioresour. Technol..

[30] Hemwimol S., Pavasant P., Shotipruk A. (2006). Ultrasound-assisted extraction of anthraquinones from roots of *Morinda citrifolia*. Ultrason. Sonochem..

[31] Chan E., Lim Y., Wong S., Lim K., Tan S., Lianto F., Yong M. (2009). Effects of different drying methods on the antioxidant properties of leaves and tea of ginger species. Food Chem..

[32] Silva E., Rogez H., Larondelle Y. (2007). Optimization of extraction of phenolics from *Inga edulis* leaves using response surface methodology. Sep. Purif. Technol..

[33] Liyana-Pathirana C., Shahidi F. (2005). Optimization of extraction of phenolic compounds from wheat using response surface methodology. Food Chem..

[34] Lissi E.A., Modak B., Torres R., Escobar J., Urzua A. (1999). Total antioxidant potential of resinous exudates from *Heliotropium* species, and a comparison of the ABTS and DPPH methods. Free Radic. Res..

[35] Wang M., Li J., Rangarajan M., Shao Y., LaVoie E.J., Huang T.C., Ho C.T. (1998). Antioxidative phenolic compounds from sage (*Salvia officinalis*). J. Agric. Food Chem..

[36] Prior R.L., Wu X., Schaich K. (2005). Standardized methods for the determination of antioxidant capacity and phenolics in foods and dietary supplements. J. Agric. Food Chem..

[37] Mustafa A., Turner C. (2011). Pressurized liquid extraction as a green approach in food and herbal plants extraction: A review. Anal. Chim. Acta.

[38] Garcia-Castello E., Rodriguez-Lopez A., Mayor L., Ballesteros R., Conidi C., Cassano A. (2015). Optimization of conventional and ultrasound assisted extraction of flavonoids from grapefruit (*Citrus paradisi* L.) solid wastes. LWT-Food Sci. Technol..

[39] Luthria D.L., Biswas R., Natarajan S. (2007). Comparison of extraction solvents and techniques used for the assay of isoflavones from soybean. Food Chem..

[40] Alothman M., Bhat R., Karim A. (2009). Antioxidant capacity and phenolic content of selected tropical fruits from Malaysia, extracted with different solvents. Food Chem..

[41] Otero-Pareja M.J., Casas L., Fernández-Ponce M.T., Mantell C., Ossa E.J. (2015). Green extraction of antioxidants from different varieties of red grape pomace. Molecules.

[42] European Food Safety Authority (2012). Scientific Opinion on the evaluation of the substances currently on the list in the annex to Commission Directive 96/3/EC as acceptable previous cargoes for edible fats and oils. Part II of III. EFSA J..

[43] The Joint FAO/WHO Expert Committee on Food Additives Toxicological evaluation of some extraction solvents and certain other substances. The FAO Nutrition Meetings Report Series 48A, Proceedings of the Fourteenth Meeting of the Joint FAO/WHO Expert Committee on Food Additives.

[44] Butnariu M. (2012). An analysis of Sorghum halepense’s behavior in presence of tropane alkaloids from *Datura stramonium* extracts. Chem. Cent. J..

[45] Thoo Y.Y., Ho S.K., Liang J.Y., Ho C.W., Tan C.P. (2010). Effects of binary solvent extraction system, extraction time and extraction temperature on phenolic antioxidants and antioxidant capacity from mengkudu (*Morinda citrifolia*). Food Chem..

[46] Zhang Z.S., Li D., Wang L.J., Ozkan N., Chen X.D., Mao Z.H., Yang H.Z. (2007). Optimization of ethanol-water extraction of lignans from flaxseed. Sep. Purif. Technol..

[47] Yuan J., Huang J., Wu G., Tong J., Xie G., Duan J.A., Qin M. (2015). Multiple responses optimization of ultrasonic-assisted extraction by response surface methodology (RSM) for rapid analysis of bioactive compounds in the flower head of *Chrysanthemum morifolium* Ramat. Ind. Crops Prod..

[48] Shin Y., Liu R.H., Nock J.F., Holliday D., Watkins C.B. (2007). Temperature and relative humidity effects on quality, total ascorbic acid, phenolics and flavonoid concentrations, and antioxidant activity of strawberry. Postharvest Biol. Technol..

[49] Ebrahimzadeh M.A., Pourmorad F., Bekhradnia A.R. (2008). Iron chelating activity, phenol and flavonoid content of some medicinal plants from Iran. Afr. J. Biotechnol..

[50] Tay P.Y., Tan C.P., Abas F., Yim H.S., Ho C.W. (2014). Assessment of extraction parameters on antioxidant capacity, polyphenol content, epigallocatechin gallate (EGCG), epicatechin gallate (ecg) and iriflophenone 3-C-β-glucoside of agarwood (*Aquilaria crassna*) young leaves. Molecules.

[51] Zhou J., Zheng X., Yang Q., Liang Z., Li D., Yang X., Xu J. (2013). Optimization of ultrasonic-assisted extraction and radical-scavenging capacity of phenols and flavonoids from *Clerodendrum cyrtophyllum* turcz leaves. PLoS ONE.

[52] Liu Z.C., Zhang L.K., Yu C., Feng J.G. (2012). Optimization of ultrasound assisted extraction of flavonoids from germinated brown rice using response surface methodology. Food Sci..

[53] Ruiz-Gutiérrez M.G., Amaya-Guerra C.A., Quintero-Ramos A., Pérez-Carrillo E., Ruiz-Anchondo T.J., Báez-González J.G., Meléndez-Pizarro C.O. (2015). Effect of extrusion cooking on bioactive compounds in encapsulated red cactus pear powder. Molecules.

[54] Wang S.P., Dong X.F., Tong J.M. (2013). Optimization of enzyme-assisted extraction of polysaccharides from alfalfa and its antioxidant activity. Int. J. Biol. Macromol..

[55] Thoo Y.Y., Ho S.K., Abas F., Lai O.M., Ho C.W., Tan C.P. (2013). Optimal binary solvent extraction system for phenolic antioxidants from mengkudu (*Morinda citrifolia*) fruit. Molecules.

[56] Paz M., Gúllon P., Barroso M.F., Carvalho A.P., Domingues V.F., Gomes A.M., Becker H., Longhinotti E., Delerue-Matos C. (2015). Brazilian fruit pulps as functional foods and additives: Evaluation of bioactive compounds. Food Chem..

